# Supplementary material for: Pharmacological and In Silico Analysis of Oat Avenanthramides as EGFR Inhibitors: Effects on EGF-Induced Lung Cancer Cell Growth and Migration
Source: Int J Mol Sci. 2022 Aug 1;23(15):8534. doi: 10.3390/ijms23158534 (PMC9369115; doi:10.3390/ijms23158534)

Figure S1A. Representative images of clonogenic assay reported in figure 3 of the main text

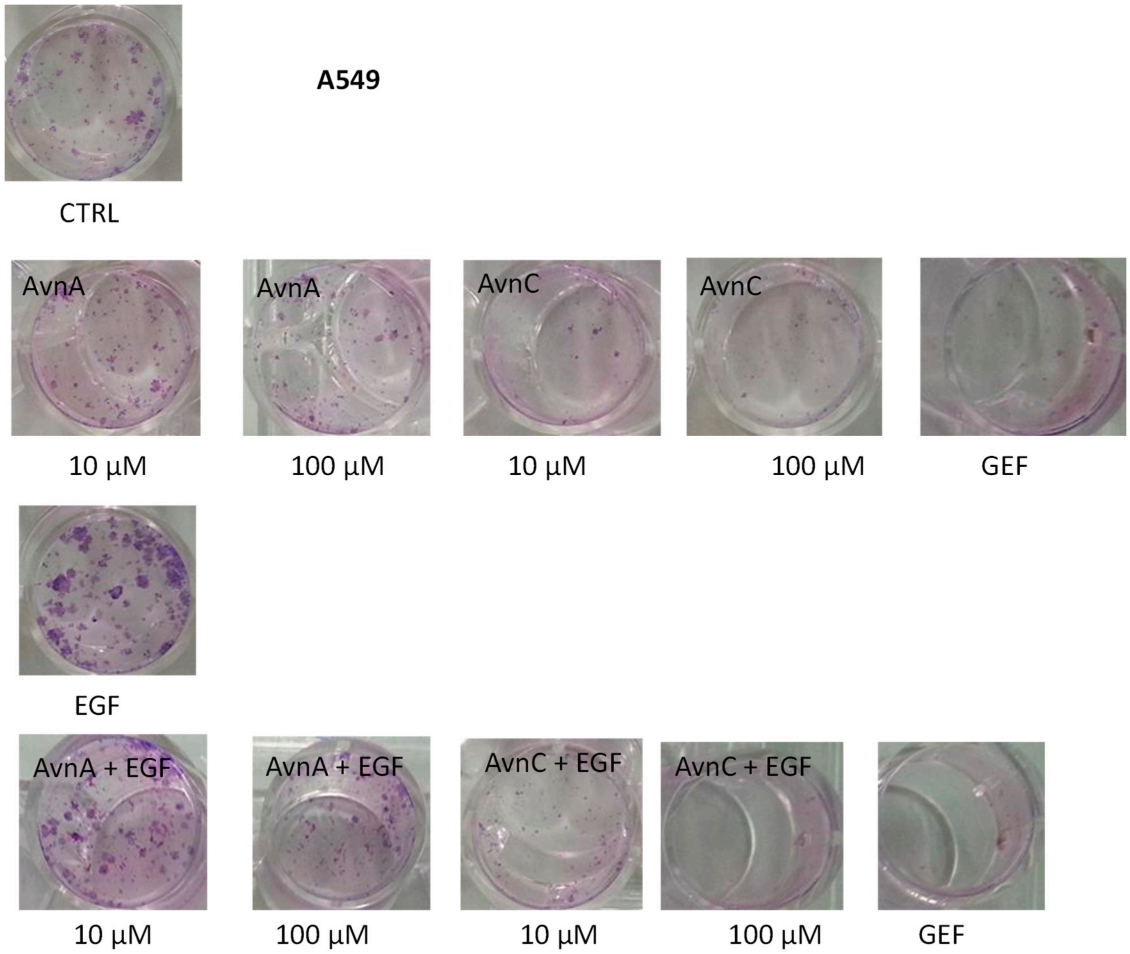

Figure S1B. Representative images of clonogenic assay reported in figure 3 of the main text

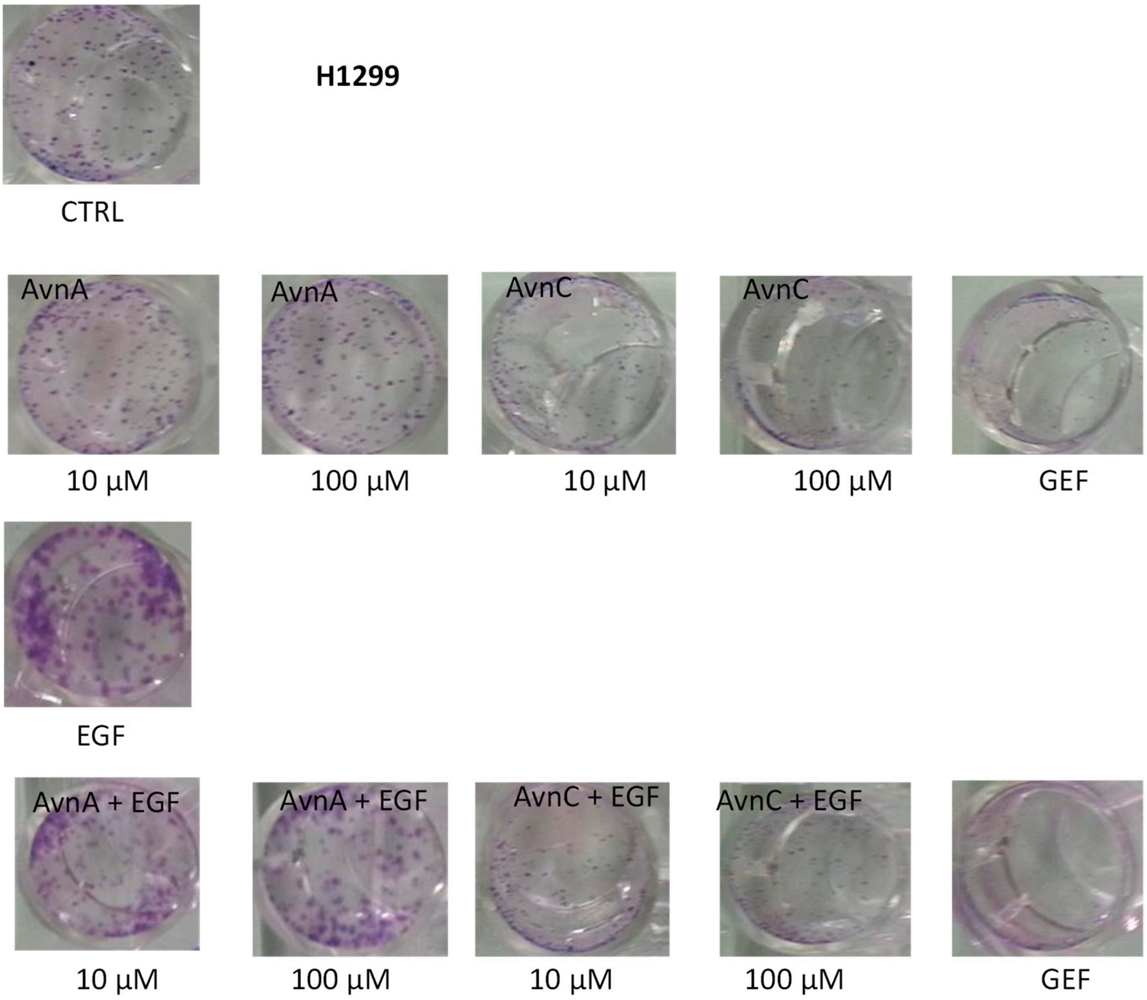

Figure S2. Avns reduce migration of tumor cells promoted by EGF. Representative images of A549 migration evaluated by scratch assay.

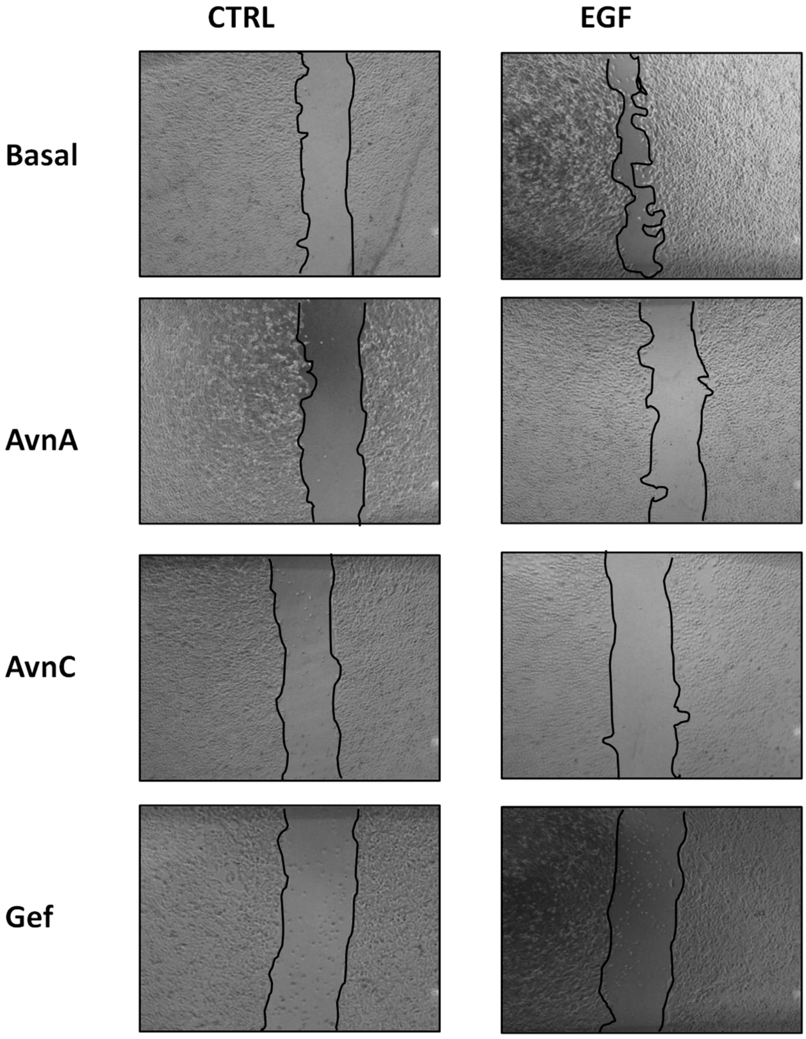

Figure S3. Raw data of Figure 5

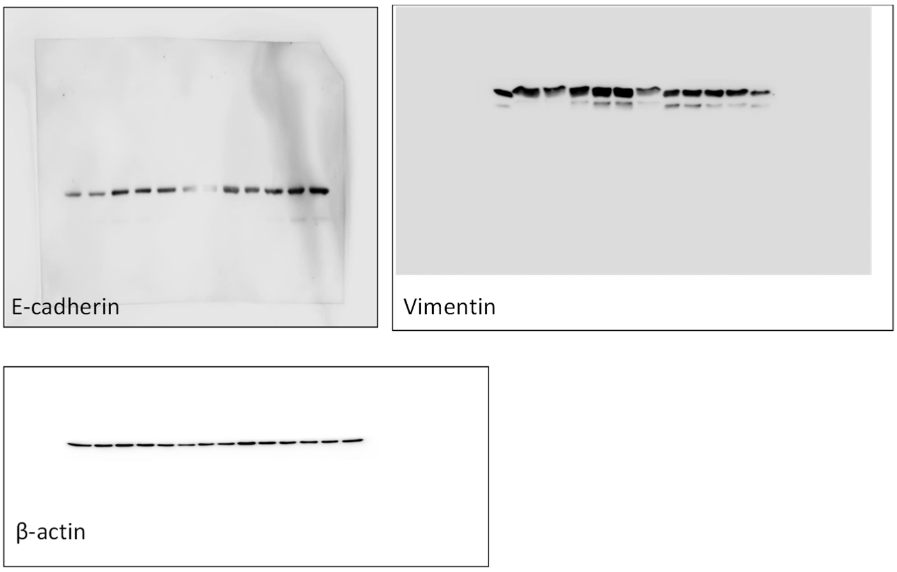

Figure S4. Raw data of Figure 6

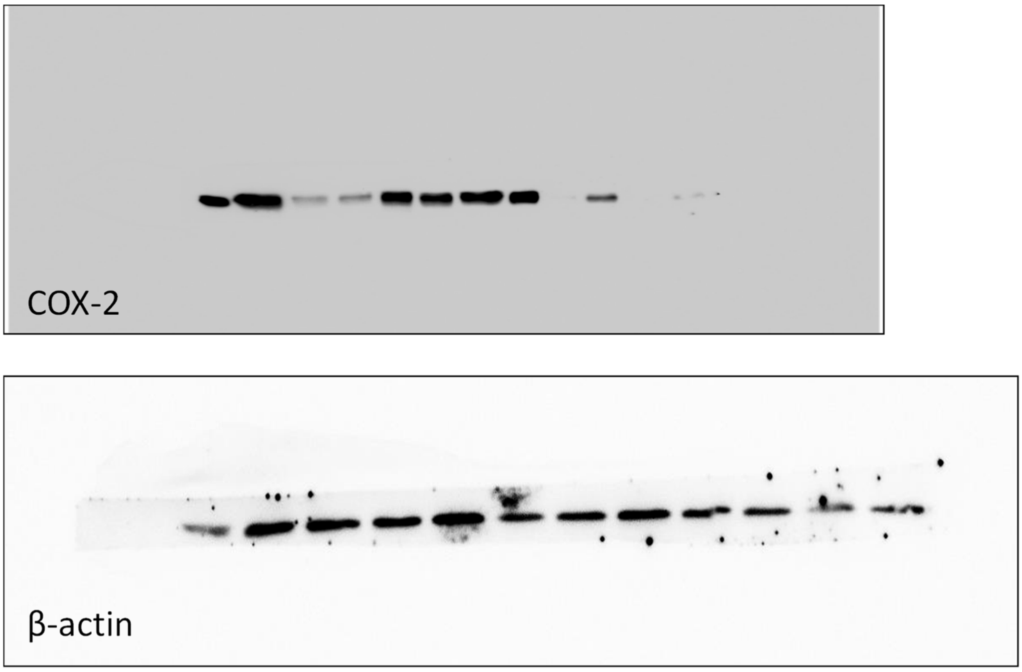

Figure S5. Raw data of Figure 7

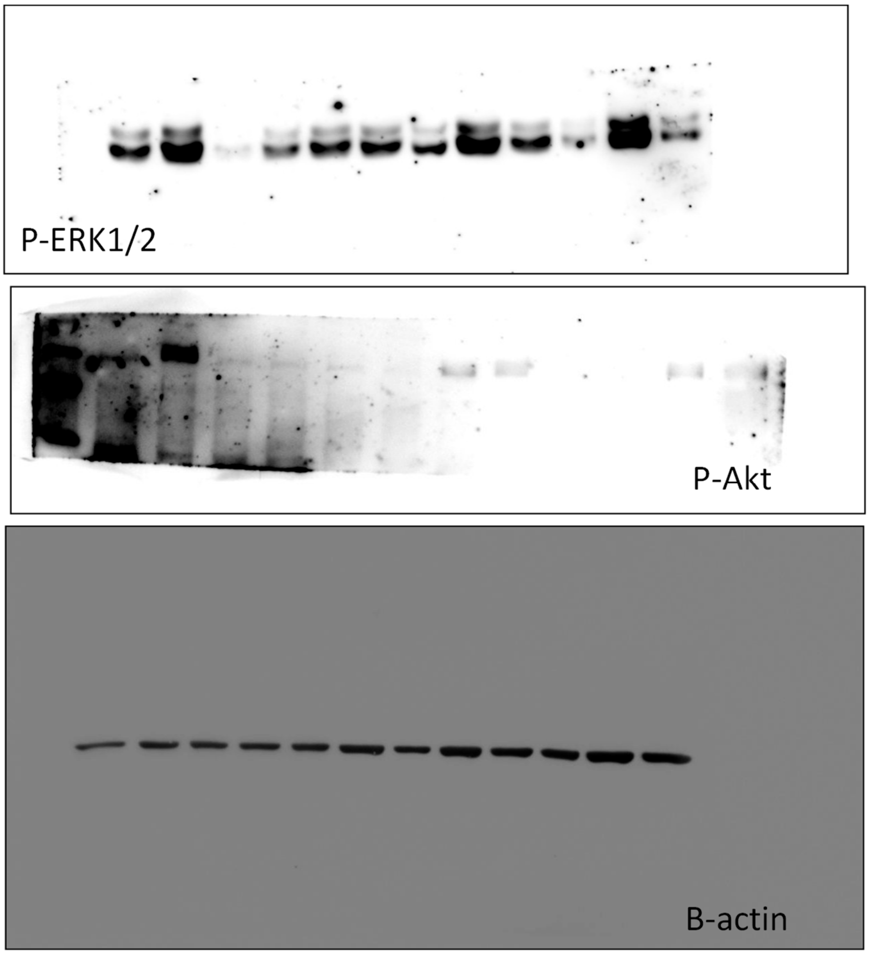

Figure S6. Raw data of Figure 8

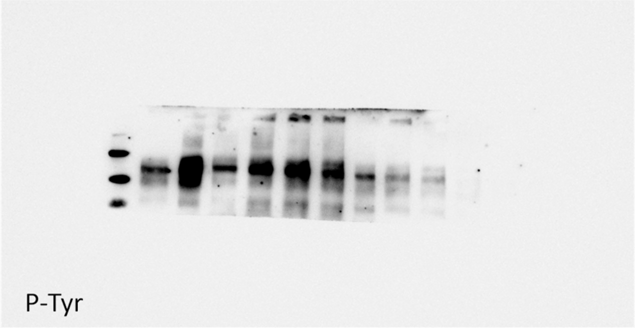

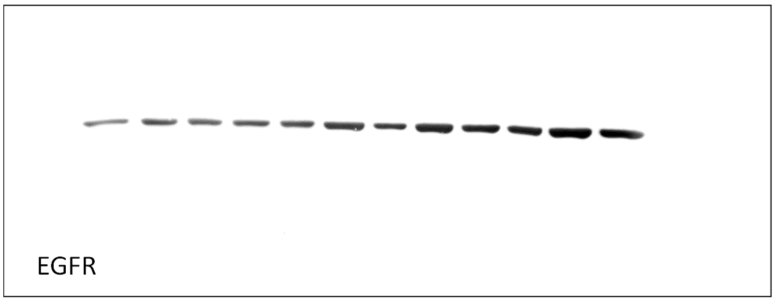

Figure S7. Raw data of Figure 9B

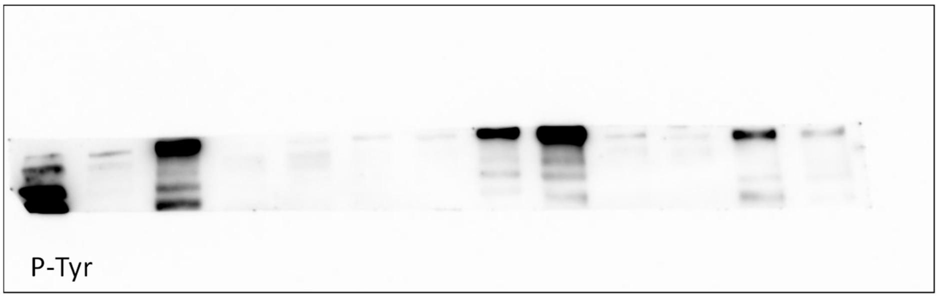

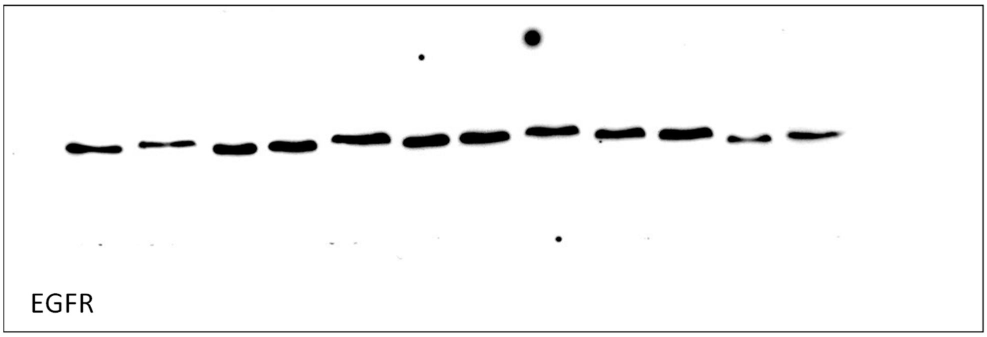

Figure S8. Raw data of Figure 9C

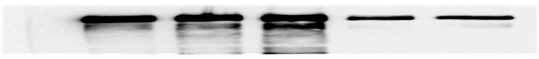

P-Tyr

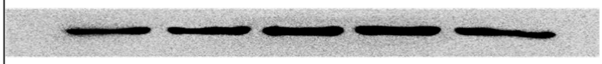

EGFR

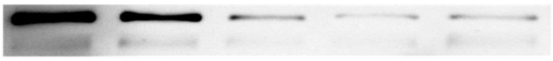

P-Tyr

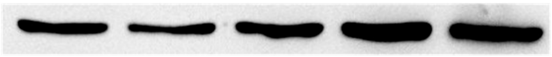

EGFR

Figure S9. Overview of gefitinib docked within the EGFR binding pocket. Gefitinib is reported as grey sticks. The residues involved in hydrogen/halogen bonds (blue and yellow continue lines), salt bridge (red dotted line), and hydrophobic interactions (grey dotted line) are indicated as green, brown, orange, and violet, sticks/balls, respectively. The yellow sphere represents the charge centre of atoms forming the salt bridge. To clarify, the hydrogens were hidden.

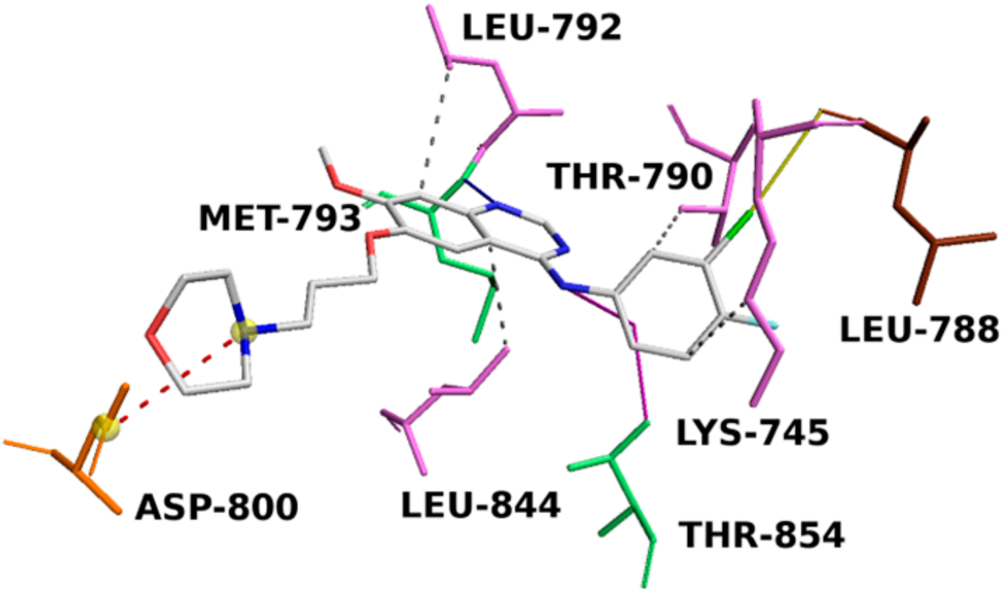

Figure S10. Root Mean Square Deviation (RMSD) plot. EGFR in complex with AvnA, AvnC, and gefitinib and the binding pose of compounds are shown as cyan, purple, and grey lines, respectively. RMSD profile of protein backbone of EGFR in complex with compounds and the docked pose in complex with target binding pocket are represented as continue and dotted lines. Molecular dynamics run time (100000 ps) is reported on the X-axis, while RMSD values (nm) are shown on the Y-axis.

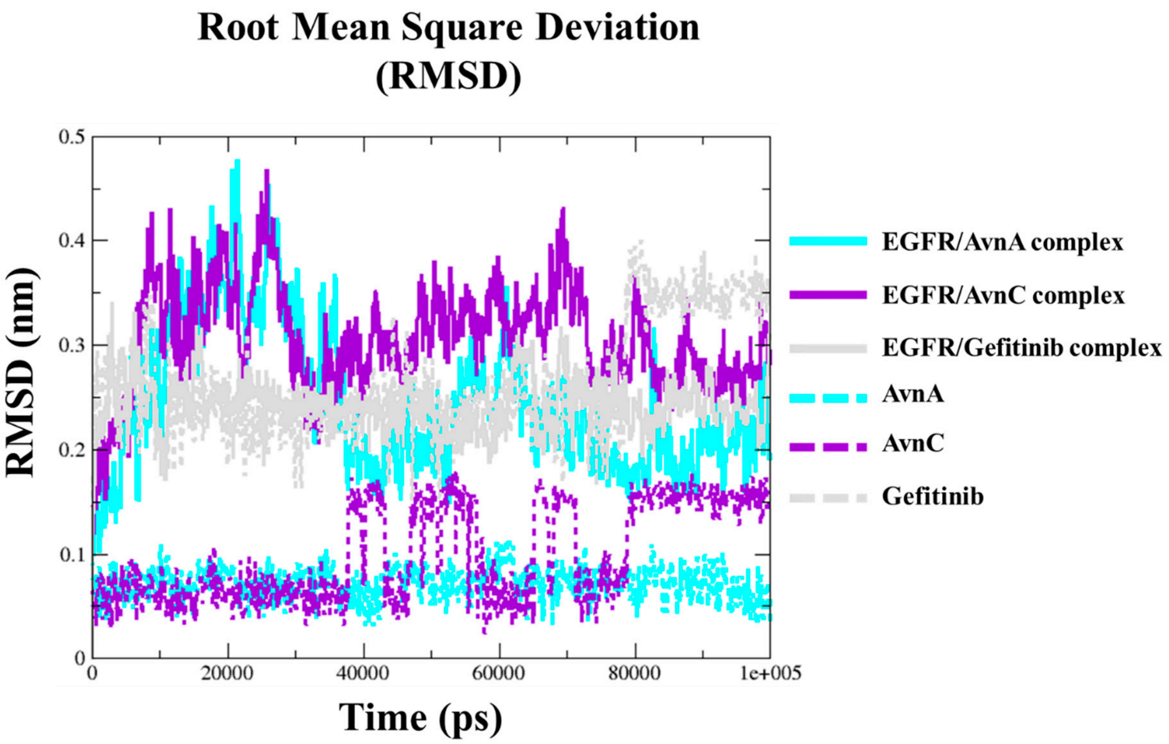

Video S1. Molecular dynamics simulation (100 ns) of EGFR-Water-Avns water bridge lifetime. H-bond existence trigged between either (A) AvnA (cyan sticks) or (B) AvnC (purple sticks) with water molecule (the hydrogen and oxygen atoms were represented as white sticks and gold sphere, respectively) and EGFR Thr-854 binding residue (in green sticks). Hydrogen bonds are depicted as a blue dashed line. To clarify, EGFR protein and all water molecules were hidden. The trajectories and Video were visualized and generated in PyMOL 2.3.

Figure S11. Root Mean Square Fluctuation (RMSF) plot. The RMSF trends of EGFR in complex with AvnA, AvnC, and Gefitinib are shown as cyan, purple, and grey lines, respectively. The Enlargement reports a zoom in of the αC helix region and DFG motif. The EGFR residues are reported on the X-axis, while RMSF values (nm) are shown on the Y-axis.

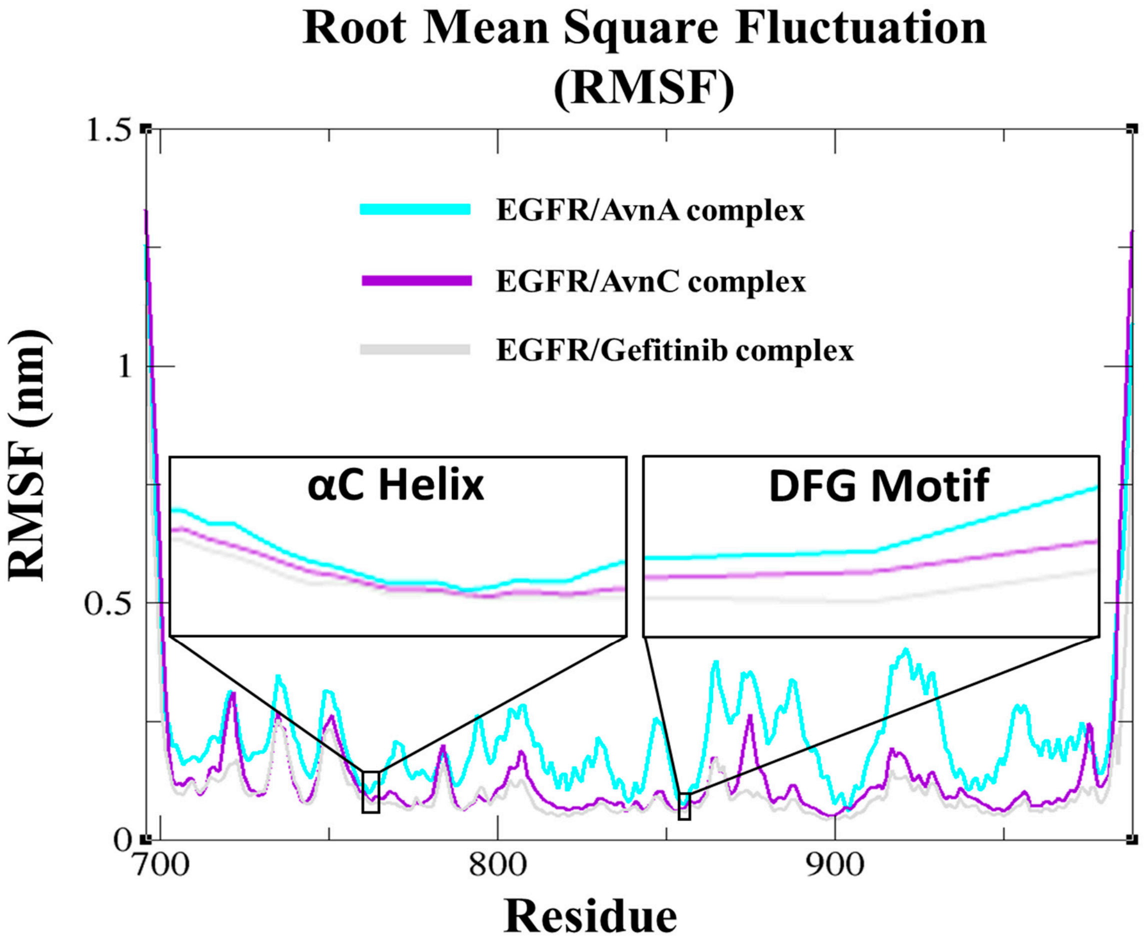

Figure S12. Interaction energy plot. The Short-Range Lennard-Jones (LJ-SR) and Coulomb (Coul-SR) interaction energy trends of EGFR in complex with AvnA, AvnC, and Gefitinib are shown as cyan, purple and grey line and dotted-lines, respectively. The interaction energy is reported on the Y-axis (kJ/mol), while the MD run time (ps) is shown on the X-axis.

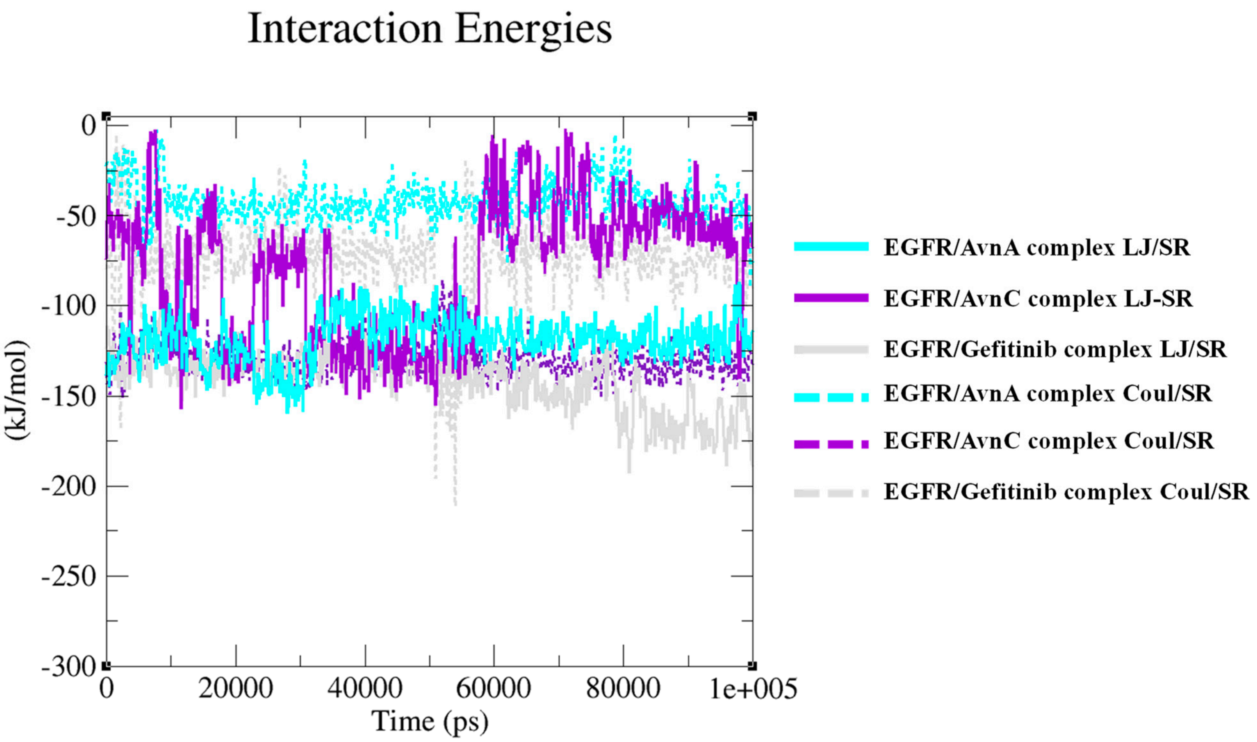

Supplement: Supplementary file 1 [file ijms-23-08534-s001.zip › ijms-1828601-supplementary.pdf]
